# Verocytotoxin-producing *Escherichia coli*, Japan, 1999–2004

**DOI:** 10.3201/eid1202.050268

**Published:** 2006-02

**Authors:** Mio Sakuma, Mitsuyoshi Urashima, Nobuhiko Okabe

**Affiliations:** * Jikei University School of Medicine, Tokyo, Japan;; † National Institute of Infectious Diseases, Tokyo, Japan

**Keywords:** Escherichia coli, 0157, emerging infectious disease, epidemiology, child, dispatch

## Abstract

In 1999, an infectious disease prevention law was enacted in Japan that affected the nationwide infectious surveillance system. A total of 19,304 laboratory-confirmed verocytotoxin-producing *Escherichia coli* cases were reported through 2004. The annual incidence was 2.74/100,000 population; its fluctuation over time and space was associated with climate, socioeconomic, and population factors.

Triggered by 2 major outbreaks of verocytotoxin-producing *Escherichia coli* (VTEC) in Japan (*1**,**2*), the nationwide surveillance system of the National Institute of Infectious Diseases (NIID) was reengineered in April 1999 by enacting a new infectious disease prevention law to better ascertain the state of laboratory-confirmed VTEC cases across the nation. In this study, we used these nationwide, population-based surveillance data to determine the infectious status of VTEC and to explore factors that affect the incidence of VTEC.

## The Study

Since the new surveillance system under the new law began, all laboratory-confirmed VTEC cases are reported and counted in Japan. Under this system, stool samples or rectal swabs are obtained from patients when the clinician suspects hemorrhagic enterocolitis due to pathogenic *E. coli* based on clinical symptoms such as hemorrhagic colitis. These specimens are sent to laboratories at the hospital, private companies, national institutions in each prefecture, or the NIID. To maintain high levels of sensitivity and specificity to detect VTEC, the protocol and training in these laboratories fall under the guidance of the NIID. At these laboratories, the specimens are cultured on specific media such as CHROMagar O157 (Kanto Co. Ltd., Tokyo, Japan) or cefixime-tellurite sorbitol MacConkey agar (Oxoid, Unipath Ltd., Hampshire, UK); specific antibodies against each serotype of *E. coli* are used (*3**,**4*). If the existence of pathogenic *E. coli* is confirmed, the ability to produce verocytotoxin from isolates is investigated by using reversed passive latex agglutination or a multiplex polymerase chain reaction assay (*3**,**5**,**6*).

If the production of verocytotoxin is confirmed by the laboratory, the case is considered symptomatic VTEC. Persons associated with the initial case (e.g., family members) may be further examined for VTEC at the doctor's discretion. When a doctor diagnoses either symptomatic or asymptomatic VTEC infection, he or she has to report this event to the local health center immediately and manage the cases to prevent further spread of the disease. The number of VTEC cases in Japan is totaled for each prefecture weekly. In this study, we used this surveillance data reported from April 1999 to October 2004 (287 weeks), which were retrieved from the Infectious Agents Surveillance Report published by NIID. Climate variables, which were summarized weekly, were retrieved from meteorologic agencies in the capitals of the 47 prefectures. Considering the incubation period between infection and reporting a diagnosis of VTEC, we used the climatic conditions from the 2-week period before each case was reported. We also used annual socioeconomic data for each of the 47 prefectures (*7*), including the following information: population density, percentage of children (<15 years of age), percentage of elderly (>65 years of age), average number of persons in the household, number of livestock (beef cattle, dairy cattle, hogs, and chickens) per person in the prefecture, and average income. All statistical analyses were performed by using Stata 8.0 software (Stata Corp. LP, College Station, TX, USA).

## Conclusions

Nationwide, 19,304 cases of VTEC were reported during the study. The annual incidence was 2.74 per 100,000. The highest number that occurred in a prefecture was 63 VTEC cases per 1,000,000 during 1 week in a single prefecture. More than 16 VTEC cases were observed in 10% of 13,489 weeks (287 weeks × 47 prefectures), and no cases were reported in 57.6% of 13,489 weeks. Age distribution of patients indicates that the number of VTEC cases was highest in children <5 years of age and fewer cases were reported in older age groups. A total of 65 outbreaks, defined as >11 laboratory-confirmed VTEC cases in a certain time frame and area, were reported during the study. The biggest outbreak occurred during September 2003 in Kanagawa prefecture and included 252 symptomatic and 197 asymptomatic cases of VTEC.

**Figure 1:**
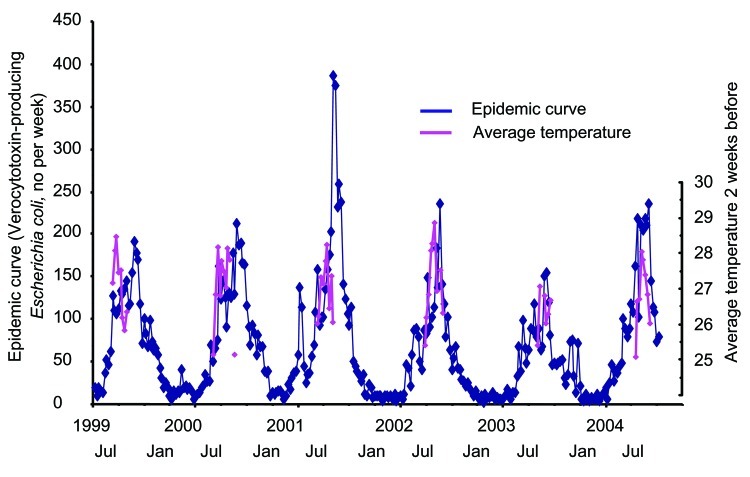
The annual oscillation of verocytotoxin-producing *Escherichia coli* (VTEC) cases during the study period. In addition to the VTEC cases, the average air temperature (>25°C) during each week of the summer season is overlaid in the graph.

The change in VTEC cases over time is shown in Figure 1, on which the average air temperature (>25°C) during each week of the summer season is overlaid. Though the annual incidence showed no clear tendency to increase or decrease during this study, a marked seasonal oscillation pattern with peaks centered in July and August was shown.

**Figure 2:**
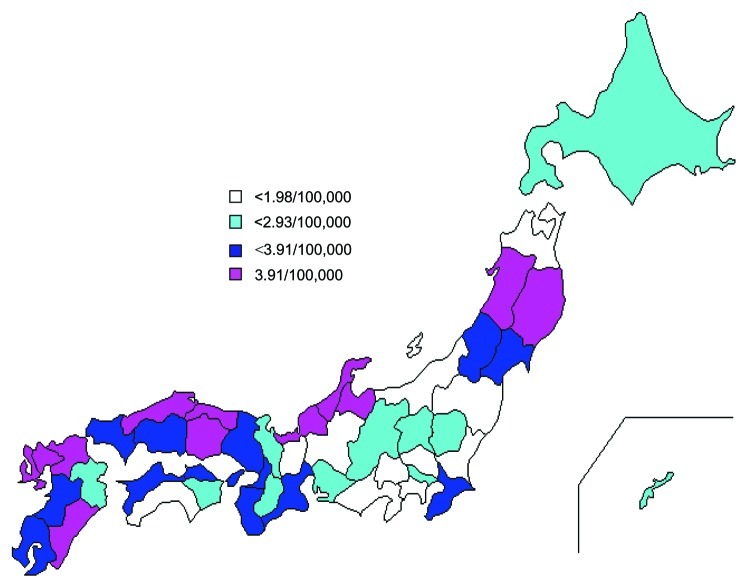
Average number of VTEC cases per 100,000 population per year in each of 47 prefectures from 1999 to 2004, Japan.

The geographic distribution of VTEC cases per 100,000 per year in each of the 47 prefectures indicated that a relatively higher incidence of VTEC was clustered in western sections of several Japanese prefectures and northeastern sections of 2 Japanese prefectures (Figure 2). The 4 prefectures with the highest annual incidences were rural areas: Saga (9.2/100,000), Ishikawa (7.9/100,000), Akita (5.8/100,000), and Iwate (5.8/100,000). Conversely, the prefectures with the lowest incidences were near urban areas: Yamanashi (1.3/100,000), Ibaraki (1.1/100,000), Niigata (0.9/100,000), and Shizuoka (1.4/100,000).

The association of climate and socioeconomic factors with the fluctuation of VTEC cases was estimated by using multiple regression analyses (Table). Within the climate variables, average air temperature of the day, wind speed, and the number of sunny days were significantly associated with the incidence of VTEC cases per 100,000 per week per prefecture. By adjusting for these 3 climate variables as well as calendar months, associations between 7 socioeconomic variables and VTEC incidence/100,000 population per week per prefecture were analyzed. Results indicated that the following population-related factors were strong risk factors for VTEC incidence: a higher percentage of elderly people in the prefecture, higher population density, higher number of persons in a household of the prefecture, and higher percentage of children. The following socioeconomic factors in the prefecture showed a positive association with VTEC incidence: lower average income in the prefecture and greater number of beef cattle per person. On the other hand, the number of chickens per person was negatively associated with VTEC incidence. Moreover, this multiple regression model showed that these population, socioeconomic, and climate factors could statistically explain 31% of the variability of VTEC incidence.

**Table:** Climate and socioeconomic variables associated with the number of cases of verocytotoxin-producing Escherichia coli* by multiple linear regression†

Climate‡ and socioeconomic§ variable	*t*	p value
Average air temperature of the day (°C)	9.72	<0.001
Wind speed (m/s)	4.69	<0.001
No. sunny days	–1.91	Not significant
Average no. persons in a household¶	6.30	<0.001
Population density	8.61	<0.001
% children (<15 years of age)	2.69	0.007
% elderly (>65 years of age)	20.70	<0.001
Average income#	–10.43	<0.001
Beef cattle/population**	2.71	0.007
Chicken/population	–3.36	0.001

*5,580 of 13,489 weeks (287 weeks × 47 prefectures) or 41.4 % of the weeks were included in the analysis as no cases of *E. coli* were reported during 7,909 weeks (58.6%). †R^2^ = 0.31: calculated based on the multiple linear regression model using the 7 socioeconomic variables, 3 climate variables, and calendar months. ‡Data for the 2-week period before the week *E. coli* was reported were used to approximate the period between infection and diagnosis. §Annual data in each prefecture were used. ¶Correlation between average no. persons in a household and population density was –0.4. #Correlation between average income and population density, average no. persons in a household, and percentage of elderly was 0.9, –0.4, and –0.6, respectively. **Beef cattle/population had a strong correlation with hog/population.

We cannot determine a causal relationship because of the nature of the ecologic study that we used in this research. However, the results imply that higher beef cattle density, higher population density, and more persons per household might increase the risk of developing VTEC infection.

Because our surveillance data were collected from different regions of Japan, we compared them on the assumption that 1) people seek care with the same frequency in all regions when they are ill, 2) doctors request stool specimens with the same frequency in all regions, and 3) laboratories test for VTEC with the same standards in all regions. Thus, some degree of observation bias may exist even under control of the law, which is a limitation of this study. In addition, the number of cases includes not only symptomatic but also asymptomatic VTEC, which may also raise the incidence rate in the Japanese surveillance system.

In conclusion, we showed a high annual incidence of VTEC of 2.74 per 100,000 that was associated with climate, socioeconomic, and population factors. However, because this was an ecologic study, further longitudinal studies are necessary to address these complicated associations.
